# Ethical Challenges Regarding Cosmetic Surgery in Patients with Body Dysmorphic Disorder

**DOI:** 10.3390/healthcare10071345

**Published:** 2022-07-20

**Authors:** Sorin Hostiuc, Oana-Maria Isailă, Mugurel Constantin Rusu, Ionut Negoi

**Affiliations:** 1 Department of Legal Medicine and Bioethics, Faculty of Dental Medicine, “Carol Davila” University of Medicine and Pharmacy, 042122 Bucharest, Romania; soraer@gmail.com; 2 “Mina Minovici” National Institute of Legal Medicine, 042122 Bucharest, Romania; 3 Department of Anatomy, Faculty of Dental Medicine, “Carol Davila” University of Medicine and Pharmacy, 020021 Bucharest, Romania; anatomon@gmail.com; 4 Department of Surgery, Faculty of Medicine, “Carol Davila” University of Medicine and Pharmacy, 020021 Bucharest, Romania; negoiionut@gmail.com

**Keywords:** plastic surgery, body dysmorphic disorder, beneficence, autonomy, loyalty

## Abstract

Body dysmorphic disorder (BDD) is an obsessive-compulsive disease, associated with increased addressability to plastic surgeons; however, as patients perceive body defects due to decreased insight, they are often unsatisfied with their appearance after cosmetic surgery. The purpose of this study is to evaluate the ethical reasoning that should be performed before accepting these patients as cosmetic surgery candidates. We will focus our analysis on three main areas of interest: autonomy, which in these patients is significantly decreased, beneficence as satisfaction, which in these particular patients does not justify performing the intervention, and loyalty, which should render cosmetic procedures immoral in patients with body dysmorphic disorder.

## 1. Introduction

Patients requesting cosmetic surgery often suffer from various psychiatric disorders, including bipolar disorder, factitious disorders, Munchausen syndrome, intentional self-harm, schizophrenia, body dysmorphic delusion, obsessive-compulsive disorders (body dysmorphic disorder—BDD), personality disorders (borderline, narcissistic, obsessive-compulsive), as well as social phobias (anxiety disorders), somatoform disorders (somatization disorders, hypochondriasis), reaction to severe stress (acute stress reaction, post-traumatic stress disorder), or adjustment disorder [1]. Of these, one of the most important is BDD; it has an overall prevalence of 1% in the US [2], while among cosmetic surgery patients it reaches 15% [3].

According to DSM-V, BDD is an obsessive-compulsive disorder, characterized by: (A) a preoccupation with one or more perceived defects or flaws in the physical appearance that are unnoticeable or appear slight to others; (B) the presence of repetitive behaviors or mental acts at some point during the course of the disorder, in response to the individual’s concerns regarding their appearance; (C) significant distress or impairment in various areas of functioning (social, occupational), caused by this preoccupation; and (D) this preoccupation with appearance not being better explained by concerns about weight or body fat in a person whose symptoms meet the criteria for an eating disorder [4].

The purpose of this article is to evaluate the ethical framework underlying ethical decision-making affecting patients with BDD requesting cosmetic intervention.

## 2. Methods

We performed an unsystematized review of the scientific literature regarding the ethical principles and moral values underlying the decision to comply with the requests of patients with BDD for cosmetic intervention. The search was performed using Web of Science, PubMed, and Google Scholar. We included peer-reviewed studies that analyzed BDD from a clinical and ethical perspective, in terms of the medical approach to this pathological phenomenon.

## 3. Results

### 3.1. Decision to Operate

Crerand et al. summarized the scientific literature regarding the psychological disorders associated with cosmetic interventions, and found that it has evolved in three main stages [5], as follows (Table 1):

The typical patient with BDD seeking cosmetic treatment is in his or her thirties, although though the appearance of the first signs often occurs at a significantly younger age [6]. Approximately equal percentages of both genders are affected [7,8], the condition is continuous rather than episodic, worsening over time, showing a low response to psychopharmacological agents, and the presence of a variable but usually decreased insight [6]. The most important signs include obsessive thoughts about the perceived defects [6] (sometimes so severe that the patient is unable to think about anything else), compulsive behaviors (such as looking in the mirror for hours to view the defects, skin picking, and using clothes or body positions to hide the perceived defects) [9], increased anxiety (causing issues in interpersonal relationships) [10], preoccupation with the perceived defects (which is variable depending on gender, with men being usually interested in their genitals, hair, or body constitution, while women are concerned with breasts, body constitution, and legs [7]), aggressive behaviors, decreased self-esteem [11,12], and suicidal ideation [6]. All these are thought by patients to be solved through cosmetic procedures (dental, dermatological, plastic surgery, etc.); moreover, as these patients sometimes request enhancement of the body part that is perceived as deficient (sometimes up to seven times during the course of their disease) [6], they often require multiple interventions, on multiple body parts, and the perceived result is often unsatisfying.

The decision to operate should be highly dependent upon the psychological status of the patient. However, neither party of the physician–patient relationship has an intrinsic motive to ask for a psychological evaluation; the surgeon is not used to routinely asking for it before elective surgery, while the patients are often “in the dark” regarding a potential psychiatric disorder characterized by a need for bodily enhancements through cosmetic procedures [1]. Moreover, physicians who request a psychological evaluation are at risk of losing patients who may not be willing to accept a psychiatric consultation before a rhinoplasty, or a breast augmentation, for example. Such a request might be seen as demeaning and/or a way of increasing the overall cost of the procedure, further decreasing its economic desirability for the physician due to the cost increase associated with intervention. However, performing plastic surgery on a BDD patient could lead to dissatisfaction, might exacerbate the underlying psychiatric condition, or increase the litigability of the physician [1].

For these reasons, various authors have developed algorithms for screening patients with high risk of BDD, which can be used by any plastic surgeon. For example, according the NICE guidance regarding the diagnosis of BDD, there are five essential questions which the prospective patient should answer, namely: (1) “Do you worry a lot about the way you look, and wish you could think about it less?”; (2) “What specific concerns do you have about your appearance?”; (3) “On a typical day, how many hours a day is it on your mind?” (more than 1 h is excessive); (4) “What effect does it have on your life?”; (5) “Does it make it hard to do your work or be with your friends?” [13]. Oosthuizen, Lambert and Castle developed a seven item questionnaire (Dysmorphic Concerns Questionnaire), each with four potential answers [14]. Dufresne et al. developed a specific questionnaire to screen dermatology patients for BDD [15]. Veale et al. identified nine items which could differentiate cosmetic surgery candidates with BDD from candidates without it [16]. Harth and Hermes developed an algorithm for evaluating the usefulness of surgery in a patient with psychosomatic disturbances. According to their method, possible indications for surgery include: no mental disorder, a high degree of psychological strain (internal resistance), an objective physical defect, realistic expectations, feasibility of surgery, and expected improvement after surgery; contraindications for surgery include: mental disturbances, no objective physical deformity, BDD, suicidal tendencies, unrealistic expectations, multiple unsuccessful corrective surgical interventions, unacceptable risks associated with surgery, and impeding aggravation [1].

### 3.2. Phronesis and Patient Self-Perception

The perception of one’s own body consists of constitutive elements as well as subjective elements transposed into experience. According to Fuchs, one can discuss a more complex dualism than the body/mind, the precept of the constitutive body being more pronounced in situations where there are signs of fatigue, physical tasks, or pathological conditions; situations in which the person does not show the necessary skill, the body being compared to a tool that is difficult to manage; and situations in which the person is analyzed or examined by other people [17].

BDD is characterized by exaggerated fears concerning alleged bodily ugliness. Feelings such as intense shame, fear of exposure, and fear of being looked at, increase in the presence of others and attenuate when the person is alone, contributing to phenomena of withdrawal and social isolation [17]. The study conducted by Kaplan et al. associated BDD with a perceptual instability of body image and aberrant visual processing, and brought to the fore the need to address the paradigms of sensory integration in potential therapeutic approaches [18]. Given the person’s unfavorable perception of their own body, when presented in this regard to the physician for cosmetic procedures, the surgeon is faced with assessing the cosmetics of the body segment that the patient is not satisfied with, and transposing the subjective perception of the patient into satisfaction. We can hypothetically discuss a more complex therapeutic approach, which requires primarily a satisfactory mental image (an element that depends strictly on the patient’s self-perception) and also includes the modeling of the anatomical region concerned (an element that depends on the plastic surgeon). These two desiderata are interconnected, and patients with BDD shift their standards of aesthetic perception with time. Thus, the surgeon is placed in the position of being the guardian of the patient’s ”self-perception gates”, a context in which the decision to act requires caution.

Practical wisdom, or *phronesis* in the Aristotelian concept, represents the path that ensures the right means to human wellbeing [19]. According to Kaldjian, wise clinical reasoning involves goals, concrete circumstances, virtues, deliberation, and motivation to act [20]. In the particular case of BDD, the concrete circumstances are the patient’s current pathological self-perception and are questionable, affecting the surgeon’s motivation to act.

**Figure 1:**
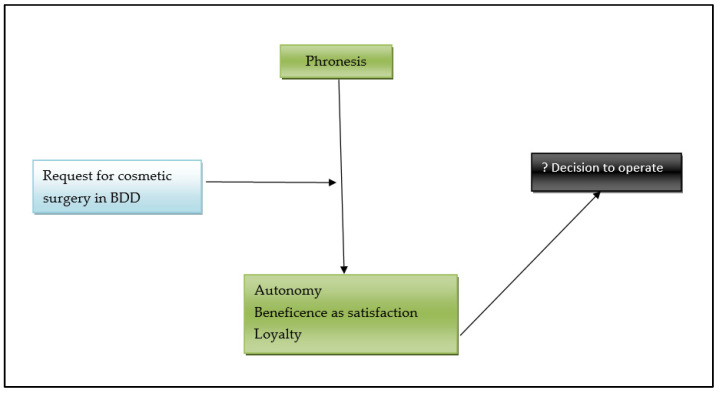
Decision to operate in a case of cosmetic procedure requested by a patient with BDD.

The elements that must be taken into account by the surgeon in the decision to act in such cases can be represented as follows (Figure 1).

### 3.3. Respect for the Autonomy of the Patient

Any patient desiring a cosmetic procedure and therefore requesting it, without a proper medical indication, has to be fully autonomous when making the decision to accept it [21,22,23], irrespective of his/her initial wishes (the determinant to accept the procedure should be volition not desire). Performing such a procedure on a patient without full autonomy is strictly forbidden, as there are no medical indications, and no way of knowing if the patient actually wants the procedure, or whether it would potentially generate some other kind of benefit for the patient (social, psychological, economic, etc.). The decision to accept the procedure must be based on proper, exhaustive information regarding the prognosis (including the expected result), benefits, risks (including the risk of not reaching the desired aesthetic result), complications, alternative corrective therapies, and financial aspects. Usually, autonomy is assessed during or after the process of informing the patient to obtain their consent—only an autonomous person can have decisional capacity to sign the consent form. Patients with BDD usually have poor insight; for example, Phillips et al. showed that among untreated patients with this disorder, 80.3% were delusional at least once during their lifetime and 47.5% were delusional during the interview, while within treated patients, the percentages were 75.4% and 33.3% respectively [6]. Marazziti et al. showed that among 30 outpatients with BDD, 17 of them had poor or absent insight [24]. Hartmann et al. found more severe insight impairment in patients with BDD compared to those with anorexia nervosa, delusionality being positively associated with the severity of BDD [25]. A systematic review performed by Ruissen et al., found a positive association between the lack of decisional competence and poor insight, the latter being identified in psychiatric patients with and without psychotic components to their presentations [26].

Based on these studies, we believe that autonomy in cosmetic surgery patients with BDD should be evaluated during the initial consultation. If at least an average insight cannot be identified, any cosmetic procedure should be halted. A recent article by Spriggs and Gilliam argued an opposite position, and gave four main arguments for these autonomy-based concerns having been overrated [27]. The first is that autonomy should be based on the way a person reasons rather than the content of their choice, which only becomes relevant if the selected choice is not intelligible or shows a lack of understanding. This is true, but presents an incomplete picture; in order to validate the capacity of a person to voluntarily accept a medical procedure, we have to be sure that external influences are not significant, and that the patient has an internal resistance sufficient to allow them to resist various types of external controls [22]. Poor insight is automatically associated with decreased internal resistance, making these patients extremely vulnerable to even minor external influences. One of the main psychological factors associated with the development of BDD is the presence of intermittent or positive reinforcement of appearance characteristics and social learning [28], including include noticing the importance of looks to other individuals, and in the mass media [29] and, more recently, social media [30]. This can be viewed as significant control for patients with low internal resistance, therefore invalidating one of the main conditions for obtaining a proper informed consent (the capacity of the patient to voluntarily accept or request the procedure). The second argument is that autonomous choices should not be automatically regarded as good choices; even bad choices may be acceptable if there is some sound rationality behind them [27]. This is true in clinical medicine, but is highly debatable in cosmetic surgery. We have to remember that, at least in most EU legal systems, cosmetic surgery is associated with a duty of result, making the physician liable if the desired result has not been obtained [31,32,33]. Even when the cosmetic procedure is justified by a correctable defect, or can lead to enhancement in line with social norms, BDD patients are more likely to sue their physicians [1]; a bad choice made by the patient (who with BDD may be delusional in a high percentage of cases, as seen above) may exponentially increase the risk of a malpractice suit. The physician–patient relationship is contractual in nature, and in order to be valid, at least in cosmetic surgery, both parties should act autonomously; if the patient’s choice is not a good one, it actively puts the physician at an increased risk of litigability. The physician’s duty of result automatically increases the level of autonomy of the patient that is needed for the contractual relationship between these two parties to develop. Therefore, the threshold of limited rationality (which in clinical medicine has been seen as optional by some authors [34,35,36]) is not sufficient in cosmetic surgery; the rationality behind choosing to undergo a cosmetic intervention should be properly assessed, and any alterations should automatically be a contraindication for surgery (at least until a psychiatric evaluation contradicts the presumption of an underlying mental disorder). The third reason is that a person exercising autonomously choice can critically reflect and make decisions based on rational arguments and are able to defend their choices in relation to their own values [27]. Again, this is true but incomplete. If rational arguments are brought to support an irrational motivation for surgery (as is often the case in patients with BDD), we should not consider the argumentation as rational, but rather as delusional. Bermudez separated procedural from epistemic rationality. In procedural rationality, the subject reasons in accordance with the formal principles of logic (such as modus ponens, modus tollens, contraposition, etc.), being mostly based on inference reasoning [37]; BDD patients with poor insight might possess procedural rationality, making them seem autonomous. In epistemic rationality, reasoning is judged in relation to the norms of good reasoning, being mainly a matter of dynamic relations regarding how beliefs relate to evidence, and how they change when the structure of evidence changes [37]. BDD patients are unable to change their rationality with changing evidence—even if they suffer cosmetic changes that are successful in relation to their preoperative wishes, they are often unsatisfied, or change their focus to other body parts; they are unable to respond to critiques regarding the fact that their body is within social norms, and therefore does not need cosmetic procedures to be in accordance with them, and so on. The last argument brought by Spriggs and Gillam is that patients have “procedural independence” [38], namely that they are not subject to influences able to significantly affect their ability to reason [27]. However, as shown above, only minor influence is needed to significantly alter their ability to reason, due to low internal resistance [22], and therefore their procedural independence is either lacking or is present at very low levels.

### 3.4. Beneficence as Satisfaction

Beneficence as an ethical principle is defined as the physician’s moral obligation to do good (mainly from a medical point of view) [22]. For some medical interventions, often in the fields of plastic surgery or aesthetic dentistry, beneficence can be viewed as satisfaction [39]. This type of beneficence is based upon the utilitarian theory of morality, promoted by philosophers like Bentham [40] and Mill [41]. According to this concept, happiness or wellbeing is the ultimate desiderate at both a personal and populational level, which makes a medical procedure morally acceptable if it generates satisfaction, even if it has no direct medical benefit. A purely aesthetic rhinoplasty does not provide the patient with any medical advantage, but it does generate a positive psychological advantage by boosting self-image, which could later improve social, cultural, and even professional skills and integration, having significant positive effects for the patient. Beneficence as satisfaction is often used as the moral justification for cosmetic procedures—they generate significant non-medical benefits, which justify the potential risks associated with a medical procedure. However, the principle of beneficence as satisfaction does not properly apply to BDD patients seeking cosmetic enhancements, who are often unsatisfied by the results; mortality by suicide is higher than in the general population, while depressive symptoms and anxiety occur irrespective of the usage of cosmetic surgery [27,28,42,43].

### 3.5. Loyalty

Loyalty is one of the most important moral virtues of physicians, being defined in general terms as an inclination to act correctly toward individuals with whom we have personal relationships; in healthcare [44], one of the main aspects of loyalty is represented by the deprioritization of the interests of the physician when these enter into conflict with the legitimate interests of the patient [22]. Cosmetic surgery is a very lucrative business, and the financial well-being of the physician is highly dependent on the number of patients and the number of procedures performed. Patients with BDD are candidates for multiple surgical interventions, and therefore physicians might be inclined to overlook a potential, underlying psychiatric disorder. However, these patients can be better treated by other physicians (who can tackle the underlying psychiatric disorder), they often do not enter the physician–patient relationship as fully autonomous, and the moral justification for performing the procedure is not present (a lack of satisfaction with the corrective changes being the rule rather than an exception). Therefore, performing cosmetic procedures on BDD patients breaches the loyalty that physicians ought to have towards their patients.

## 4. Conclusions

In conclusion we have shown that cosmetic surgery for BDD patients is not prima facie morally justified; physicians should have a high degree of suspicion, and whenever a patient with suspected BDD requests a cosmetic procedure, they should refrain from performing that procedure and send the patient for a psychological evaluation. Even if this seems counterintuitive, this approach might significantly increase the overall satisfaction of the patients, decrease the litigability of the surgeon, and in time bring additional revenue due to a lower percentage of complaints (which can reach social media, where patients often give recommendations).

## Figures and Tables

**Table 1:** The three main stages of scientific literature regarding the psychological disorders associated with cosmetic surgery [5].

Studies from the 1950s and 1960s	suggesting that psychopathology was the norm in patients requesting cosmetic surgery, with mixed results regarding psychological status after intervention
Studies from the 1970s and 1980s	which rebuffed the previous conclusions, showing decreased rates of psychopathology and potential psychological improvement after intervention
Studies from the 1990s and onward	showing that almost half of patients have a formal psychiatric diagnosis, and that surgery usually has positive psychological effects

## Data Availability

Not applicable.

## References

[1] Harth W., Hermes B. (2007). Psychosomatische störungen bei kosmetischen operationen. JDDG-J. Ger. Soc. Dermatol..

[2] Bohne A., Keuthen N.J., Wilhelm S., Deckersbach T., Jenike M.A. (2002). Prevalence of symptoms of body dysmorphic disorder and its correlates: A cross-cultural comparison. Psychosomatics.

[3] Glaser D.A., Kaminer M.S. (2005). Body dysmorphic disorder and the liposuction patient. Dermatol. Surg..

[4] Phillips K.A., Wilhelm S., Koran L.M., Didie E.R., Fallon B.A., Feusner J., Stein D. (2010). Body dysmorphic disorder: Some key issues for DSM-V. Depress Anxiety.

[5] Crerand C.E., Magee L. (2013). Cosmetic and Reconstructive Breast Surgery in Adolescents: Psychological, Ethical, and Legal Considerations. Seminars in Plastic Surgery.

[6] Phillips K.A., Menard W., Fay C., Weisberg R. (2005). Demographic characteristics, phenomenology, comorbidity, and family history in 200 individuals with body dysmorphic disorder. Psychosomatics.

[7] Phillips K.A., Diaz S.F. (1997). Gender differences in body dysmorphic disorder. J. Nerv. Ment. Dis..

[8] Tyagi H., Govender A., Drummond L.M. Gender Differences in Body Dysmorphic Disorder. Proceedings of the 165th Annual Meeting of the Ameri-can Psychiatric Association.

[9] Castle D.J., Phillips K.A. (2002). Disorders of Body Image.

[10] Rosen J.C., Reiter J., Orosan P. (1995). Cognitive-behavioral body image therapy for body dysmorphic disorder. J. Consult. Clin. Psychol..

[11] Phillips K.A., Menard W., Fay C., Pagano M.E. (2005). Psychosocial functioning and quality of life in body dysmorphic disorder. Compr. Psychiatry.

[12] Kelly M.M., Brault M.E., Didie E.R., Phillips K.A. (2017). Psychosocial functioning and quality of life in body dysmorphic disorder. Body Dys-morphic Disorder: Advances in Research and Clinical Practice.

[13] NICE Obsessive-Compulsive Disorder and Body Dysmorphic Disorder: Treatment. https://www.nice.org.uk/guidance/cg31.

[14] Oosthuizen P., Lambert T., Castle D.J. (1998). Dysmorphic concern: Prevalence and associations with clinical variables. Aust. New Zealand J. Psychiatry.

[15] Dufresne R.G., Phillips K.A., Vittorio C.C., Wilkel C.S. (2001). A screening questionnaire for body dysmorphic disorder in a cosmetic dermatologic surgery practice. Dermatol. Surg..

[16] Veale D., Ellison N., Werner T.G., Dodhia R., Serfaty M.A., Clarke A. (2012). Development of a cosmetic procedure screening questionnaire (COPS) for Body Dysmorphic Disorder. J. Plast. Reconstr. Aesthetic Surg..

[17] Fuchs T. (2002). The phenomenology of shame, guilt and the body in body dysmorphic disorder and depression. J. Phenomenol. Psychol..

[18] Kaplan R.A., Enticott P.G., Hohwy J., Castle D.J., Rossell S.L. (2014). Is body dysmorphic disorder associated with abnormal bodily self-awareness? A study using the rubber hand illusion. PLoS ONE.

[19] Aristotle (1925). *Nichomachean Ethics: Book II*; Ross, W.D., Translator. http://classics.mit.edu/Aristotle/nicomachaen.2.ii.html.

[20] Kaldjian L.C. (2010). Teaching practical wisdom in medicine through clinical judgement, goals of care, and ethical reasoning. J. Med. Ethics.

[21] Berg J., Applebaum P. (2001). Informed Consent: Legal Theory and Clinical Practice.

[22] Beauchamp T.L., Childress J.F. (2001). Principles of Biomedical Ethics.

[23] Hostiuc S. (2014). Informed Consent [Consimtamantul Informat].

[24] Marazziti D., Giannotti D., Catena M., Carlini M., Dell’Osso B., Presta S., Pfanner C., Mungai F., Dell’Osso L. (2006). Insight in Body Dysmorphic Disorder with and without Comorbid Obsessive-Compulsive Disorder. CNS Spectr..

[25] Hartmann A.S., Thomas J.J., Wilson A.C., Wilhelm S. (2013). Insight impairment in body image disorders: Delusionality and overvalued ideas in anorexia nervosa versus body dysmorphic disorder. Psychiatry Res..

[26] Ruissen A.M., Widdershoven G.A.M., Meynen G., Abma T.A., van Balkom A.J. (2012). A systematic review of the literature about competence and poor insight. Acta Psychiatr. Scand..

[27] Spriggs M., Gillam L. (2016). Body dysmorphic disorder: Contraindication or ethical justification for female genital cosmetic surgery in adolescents. Bioethics.

[28] Crerand C.E., Franklin M.E., Sarwer D.B. (2006). Body dysmorphic disorder and cosmetic surgery. Plast Reconstr. Surg..

[29] Neziroglu F., Roberts M., Yaryura-Tobias J.A. (2004). A Behavioral Model for Body Dysmorphic Disorder. Psychiatr. Ann..

[30] Griffiths S., Murray S.B., Krug I., McLean S.A. (2018). The Contribution of Social Media to Body Dissatisfaction, Eating Disorder Symptoms, and Anabolic Steroid Use Among Sexual Minority Men. Cyberpsychol. Behav. Soc. Netw..

[31] Marchesi A., Fasulo F.C., Morini O., Vaienti L. (2012). Mammaplasties and medicolegal issues: 50 cases of litigation in aesthetic surgery of the breast. Aesthetic Plast. Surg..

[32] Mangu F.I. (2010). Malpraxisul Medical. Răspunderea Civilă Medicală.

[33] Hostiuc S., Buda O. (2018). The Age of Informed Consent: A European History.

[34] Sturman E. (2005). The capacity to consent to treatment and research: A review of standardized assessment tools. Clin. Psychol. Rev..

[35] Appelbaum P.S. (2007). Assessment of Patients’ Competence to Consent to Treatment. N. Engl. J. Med..

[36] Appelbaum P.S., Roth L.H. (1983). Patients who refuse treatment in medical hospitals. JAMA.

[37] Bermudez J.L. (2003). Normativity and Rationality in Delusional Psychiatric Disorders. Mind Lang..

[38] Dworkin G. (2008). The Theory and Practice of Autonomy, Transferre.

[39] Jonsen A.R., Siegler M., Winslade W.J. (2015). A Practical Approach to Ethical Decisions in Clinical Medicine, 8E. Clinical Ethics.

[40] Bentham J., Bowring J. (1843). The Works of Jeremy Bentham.

[41] Mill J.S. (2002). The Basic Writings of John Stuart Mill: On Liberty, the Subjection of Women, and Utilitarianism.

[42] Heyes C.J. (2009). Diagnosing culture: Body dysmorphic disorder and cosmetic surgery. Body Soc..

[43] de Brito M.J.A., Xerfan N.F., Ferreira L.M. (2012). Should Plastic Surgeons Operate on Patients Diagnosed with Body Dysmorphic Disorder?. Plast. Reconstr. Surg..

[44] Pellegrino E.D., Thomasma D.C. (1993). The Virtues in Medical Practice.

